# *Drosophila king tubby (ktub)* mediates light-induced rhodopsin endocytosis and retinal degeneration

**DOI:** 10.1186/1423-0127-19-101

**Published:** 2012-12-10

**Authors:** Shu-Fen Chen, Yu-Chen Tsai, Seng-Sheen Fan

**Affiliations:** 1 Department of Life Science, Tunghai University, No. 181, Sec. 3, Taichung-Port Road, Taichung, R.O.C 407, Taiwan

**Keywords:** King-tubby, Rhabdomere, Phototransduction, Endocytosis, Retinal degeneration

## Abstract

**Background:**

The *tubby* (*tub)* and *tubby-like protein (tulp)* genes encode a small family of proteins found in many organisms. Previous studies have shown that TUB and TULP genes in mammalian involve in obesity, neural development, and retinal degeneration. The purpose of this study was to investigate the role of *Drosophila king tubby (ktub)* in rhodopsin 1 (Rh1) endocytosis and retinal degeneration upon light stimulation.

**Results:**

*Drosophila ktub* mutants were generated using imprecise excision. Wild type and mutant flies were raised in dark or constant light conditions. After a period of light stimulation, retinas were dissected, fixed and stained with anti-Rh1 antibody to reveal Rh1 endocytosis. Confocal and transmission electron microscope were used to examine the retinal degeneration. Immunocytochemical analysis shows that Ktub is expressed in the rhabdomere domain under dark conditions. When flies receive light stimulation, the Ktub translocates from the rhabdomere to the cytoplasm and the nucleus of the photoreceptor cells. Wild type photoreceptors form Rh1-immunopositive large vesicles (RLVs) shortly after light stimulation. In light-induced *ktub* mutants, the majority of Rh1 remains at the rhabdomere, and only a few RLVs appear in the cytoplasm of photoreceptor cells. Mutation of *norpA* allele causes massive Rh1 endocytosis in light stimulation. In *ktub* and *norpA* double mutants, however, Rh1 endocytosis is blocked under light stimulation. This study also shows that *ktub* and *norpA* double mutants rescue the light-induced *norpA* retinal degeneration. Deletion constructs further demonstrate that the Tubby domain of the Ktub protein participates in an important role in Rh1 endocytosis.

**Conclusions:**

The results in this study delimit the novel function of Ktub in Rh1 endocytosis and retinal degeneration.

## Background

The *tubby* (*tub)* and *tubby-like protein (tulp)* genes encode a small family of proteins found in many organisms, including *Drosophila*[1], *C. elegans*[2,3], *Gallus*[4], *Arabidopsis*[5] and other plants [6]. Four members of Tubby proteins are in mammals, including TUB, TULP1, TULP2 and TULP3 [7,8]. Mutation in mouse *tubby* gene leads to photoreceptor and cochlear degeneration and adult-onset obesity [9-11]. Target deletion of *tulp1* in mice causes photoreceptor cell degeneration [12,13]. Mutation of *tulp3*^−/−^ in mice causes defects in neural tube development, increases neuronal apoptosis, and eventually leads to embryonic lethality [14]. Human mutations of TULP1 gene result in retinitis pigmentosa, an inherited disease that typically causes retinal degeneration [15-17]. Taken together, these studies suggest an important role of Tub family proteins in retinal development and maintenance. Structural analysis indicates that the C-terminus of TULP proteins are highly conserved, and contain a DNA binding domain. The N-terminus of TULP proteins contain the remnants of the transactivation domains of many transcription factors, suggesting that TULPs may act as transcription factors [18,19]. Subsequent studies have shown that TULPs bind to actin and Dynamin-1, suggesting their function in regulating vesicle transport in photoreceptor cells [20,21]. Mutation of *tulp1* in mice causes mislocalization of rhodopsin in photoreceptor cells and abnormal formation of photoreceptor synapse [22,23]. According to recent studies, Tulps have an extracellular function in which they act as phagocytosis ligands for retinal pigment epithelium [24,25]. The role of Tulp as phagocytosis ligand occurs through binding to the MerTK, a TAM receptor tyrosine kinase subfamily [26]. Together, these studies have shown important functions of Tulps in multicellular organisms. However the molecular and cellular functions of tubby family proteins remain obscure. The *Drosophila* visual system is an excellent model for studying retinal degeneration [27-29]. The *Drosophila* genome contains one gene, *king tubby (ktub)*, belonging to the *tub* gene family. Immunocytochemical study has indicated that *ktub* is expressed in the developing nervous system, suggesting its role in neural development [1]. Whether *ktub* participates in retinal degeneration and mediates phototransduction cascade remains unclear. *Drosophila* photoreceptor cells contain specialized portions of the plasma membrane, called the rhabdomeres. Each rhabdomere consists of numerous tightly packed microvilli, rhodopsin photopigments, and other components of the phototransduction cascade [30-32]. The phototransduction cascade in *Drosophila* begins with the light activation of rhodopsin (Rh1). Once activated, Rh1 binds to heterotrimeric G protein, which catalyzes the exchange of GDP for GTP on the G_α_ subunit (G_αq_). The G_αq_ subunit then activates retinal-specific phospholipase C and causes the opening of the cation-specific channels Trp and Trpl. This eventually leads to the depolarization of the photoreceptor cell and neurotransmitter release. After light activation, rhodopsin kinase and arrestin inactivate the rhodopsin activity [33-35]. Arrestin and AP-2 are critical factors for receptor into clathrin mediate endocytosis [36]. Studies in *Drosophila* have demonstrated that visual arrestin (Arr1) is essential for light-induced Rh1 internalization [37] and Arr2 is involved in rhodopsin endocytosis under certain pathological situations [38-40].

This article studies how Ktub participates in *Drosophila* phototransduction and retinal degeneration. Results show that subcellular localization of Ktub in adult photoreceptor cells is light-dependent. In *ktub* mutant, rhodopsin endocytosis is blocked under light conditions. In addition, retinal degeneration is evident in *ktub* mutant flies reared in constant light. In *norpA* mutant, massive endocytotic rhodopsin vesicles appear in the cytoplasm. However, the rhodopsin vesicles appear less in the cytoplasm of *norpA* and *ktub* double mutant. To further investigate what domain in Ktub protein is involved in rhodopsin endocytosis, this study uses deletion constructs to examine its ability to mediate endocytosis. Results show that the C-terminal Tubby domain is required for endocytosis. Taken together, these results provide new evidence showing that the Ktub protein is required for mediating rhodopsin endocytosis and retinal degeneration.

## Methods

### *Drosophila* stocks and transgenic constructions

*Drosophila melanogaster w*^*1118*^ was used as wild type. P-element insertion fly, P(GSV6)17325/SM was obtained from the Szeged *Drosophila* stock center and was used for imprecise excision to isolate *ktub* mutants. *norpA*^*33*^, *Df (2R) ED3791*, and *rh1-Gal4* were obtained from the Bloomington stock center. All flies were reared on standard corn meal agar media at 25°C in dark, 12D/12 L light or constant-light condition. The ambient light used to create light-induced condition was approximately 500 lux. To make transgenic flies that expressed full-length, N-terminus (*N-ktub*) and C-terminus (*C-ktub*) of Ktub protein, we amplified *ktub* cDNA by *PfuTurbo* DNA polymerase from EST clone, RE38560, using the following primers: *ktub* (5’-ATGTCCGGAATCAACAGTCGTAATCAG-3’, 5’-TCACTC GCAGGCTATTT TGC-3’); *N-ktub* (5’-ATGTCCGGAATCAACAGTCGTAATCAG-3’, 5’-ATTGCCGATGACATCTCCCTCGGAC-3’)*; C-ktub* (5’-ATCGACCAGTTCGTGATGC AAC-3’, 5’-TCACTCGCAGGCTATTTTGC-3’). The PCR fragments were subcloned into *pUAST-Flag* expression vector [41] to make *pUAST-Flag-ktub, pUAST- Flag- N-ktub,* and *pUAST- Flag-C-ktub*. All constructions were verified by DNA sequencing before germ-line transformation. *P-element* mediated germ-line transformation [42] produced more than three independent lines. The transgenic lines were crossed to *rh1-Gal4* for further analysis.

### Generation of *ktub* mutant fly by P-element imprecise excision

To generate *ktub* mutants, we crossed *P(GSV6)17325/SM* to a transposase expressing line (Δ2–3) to induce P-element excision. After imprecise excision, flies with white eye were crossed to *Df (2R) ED3791* to isolate the *ktub /Df (2R) ED3791* adult flies. To identify the mutation site, two primers: 5’–GGCAATTTCAATCGAATTTACC-3’ and 5’-ATCGAA GTAACTCGAAGGACCC-3’ were used to amplify the 5’ region of the *ktub* gene. A total of 200 P-excision lines were screened to obtain six lines with DNA deletion in the *ktub* gene. DNA sequence confirmed two alleles, *ktub*^*35-3*^and *ktub*^*115-4*^*,* have deleted the ATG translation start site, and these two alleles were used for further analysis.

### Antibody production and Western blotting

To generate antibody against Ktub protein, PCR was used to amplify *ktub* fragments by two primers: 5’-ATGGAGGCCTACATCCGGCAGAAGAG-3’, 5’-TCACTCGCAGGCTATTT TGCCATCGA- 3’. The PCR products were cloned into pQE-31 vector (Qiagen, Valencia, CA). After IPTG induction, a 56 kDa Ktub recombinant protein was isolated from *E. coli* and used as antigen to inject rabbit. After several boosts, the serum was collected to test its immunoreactivity. For Western blotting, adult eyes were collected and homogenized with a homogenization buffer (50 mM HEPES, 50 mM KCl, 1 mM EGTA, 1 mM MgCl_2_, 10% Glycerol) with protease inhibitors. The cell extract was then centrifuged at 1500 g for 10 min at 4°C and subjected to SDS-PAGE to separate the protein. SDS-PAGE and Western blotting were performed with slight modification from previous studies [43]. After electrophoresis, the proteins were transferred to a PVDF membrane. To perform immune blotting, the membrane was blocked with 5% non-fat milk in TBST (10 mM Tris, pH7.4, 150 mM NaCl, with 0.1% Tween20). The membrane was then incubated with anti-Ktub antiserum (1:2000) at 4°C overnight. The following day, the membrane was washed three times with TBST and then incubated with peroxidase conjugated goat anti-rabbit IgG (1:10000). After incubation with the secondary antibody, the membrane was washed and processed for chemiluminescent reaction (Milipore, Billerica MA). The signals were detected with a CCD camera (Fuji film, Japan).

### Immunohistochemistry

To perform immunohistochemical staining, dissected eyes were fixed in 4% paraformaldehyde for 20 minutes. After three washes and blocking, the eyes were incubated with primary antibody. The primary antibodies used in this study included rabbit anti-Ktub (1:500) and mouse anti-Rh1 (4C5) from Developmental Studies Hybridoma Bank (1:100). Rhodamine or FITC conjugated phalloidin (Sigma-Aldrich, St. Louis, MO), which stained the F-actin, was used to label the cell boundary. Stained eye discs were washed three times with PBST (137 mM NaCl, 2.68 mM KCl, 10 mM Na_2_HPO_4_, 1.7 mM KH_2_PO_4,_ pH7.2 with 0.2% TritonX-100), and then incubated with secondary antibodies. The secondary antibodies used in this study were conjugated with Alex 488 (Invitrogen Molecular Probes, Carlsbad, CA), Texas Red, or Cy5 (Jackson Immuno Research Lab. West Grove, PA). After three washes, eyes were mounted in a mounting medium (0.25% n-propyl gallate, 50% glycerol in PBS, pH 8.6) and examined using a Zeiss LSM 510 confocal microscope. Images were processed using Adobe Photoshop 6.0 software.

### Electron microscopy

Transmission electron microscopy was performed as previously described [44]. Flies were injected with a fixative (2% paraformaldehyde and 2% glutaraldehyde in 0.1 M cacodylate buffer). After dissection, the eyes were incubated in fixative for another 2 hours and postfixed with 2% OsO_4_ in 0.1 M cacodylate buffer at 4°C. The eyes were subjected to series dehydration with alcohol and embedded in Epox-812 (EMS). The tissues were then sectioned using a Reichert ultramicrotome and observed using a Tecnai spirit G2 (FEI) transmission electron microscope.

## Results

### Isolation of *ktub* mutants and their phenotypic analysis

**Figure 1:**
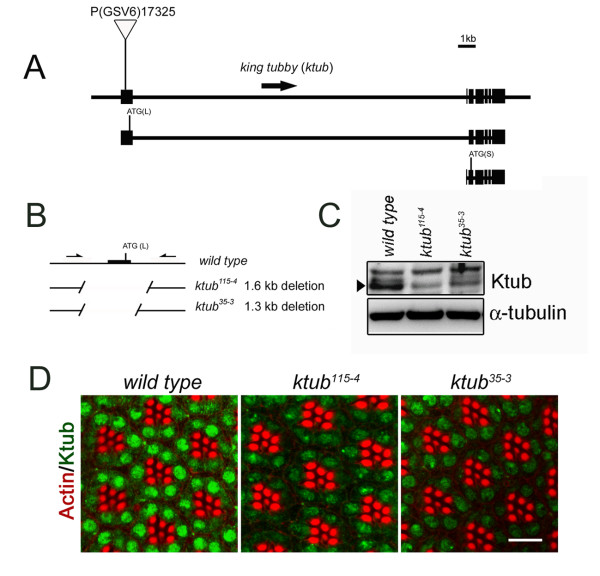
**Isolation of *ktub *mutants by P-element excision.** Schematic drawing indicates the gene structure of *Drosophila king tubby (ktub)* gene (**A**). The P-element (GSV6)17325 is inserted in the first exon of *ktub* gene. Fly genomic annotation indicates that *ktub* gene has two transcripts and two polypeptides; the long form (L) and short form (S), which are activated by distinct promoters. Arrows indicate two PCR primers, which were used to screen for the imprecise excision of *ktub* mutants (**B**). *ktub*^*115-4*^ and *ktub*^*35-3*^ mutants deleted 1.6 kb and 1.3 kb, including the translation start site of the long form transcript, respectively (**B**). Western blotting indicates the reduced of Ktub expression in mutant flies (**C**, arrowhead). Confocal images reveal that the expression of Ktub decreases significantly in mutant photoreceptor cells (**D**). Green indicates anti-Ktub antibody, and red is rhodamine-phalloidin. The scale bar is 10 μm.

Studies have shown that the mutation of human and mouse tubby family genes results in retinal degeneration, late onset obesity, and cochlear degeneration [12,13,15,45,46]. However, the biological functions of tubby genes remain obscure. Using a Basic Local Alignment Search Tool (BLAST) search, we identified *Drosophila king-tubby* (*ktub*) gene as a potential homologue of human and mouse Tubby genes. *Drosophila ktub* is located on chromosome 2R, 57B20-57C2. Genomic annotation indicates *ktub* contains two transcripts and two polypeptides. The long and the short forms of Ktub proteins are only 17 amino acids difference in their N-terminus (Additional file 1: Figure S1). This study takes the advantage of *Drosophila* genetics and uses it to study the cellular functions of Tubby proteins in phototransduction and retinal degeneration. To study the function of *ktub*, we used imprecise excision to generate *ktub* deletion mutants from P(GSV6)17325 flies (Figure 1A). Screening approximately 200 P-excision lines revealed two mutant alleles (*ktub*^*35-3*^*, ktub*^*115-4*^) whose translational initiation site ATG of long form was deleted (Figure 1B). Western blot analysis showed that Ktub expression in all mutants was significantly reduced, but not completely absent. The remained protein in the Western blot is possibly due to the expression of short form Ktub since our antibody cannot distinguish the long form and the short form of Ktub (Figure 1C). Immunocytochemistry further confirmed the Western blot data, showing that Ktub expression decreased significantly in the adult eyes of *ktub* mutants (Figure 1D). These results show that we have successfully isolated two *ktub* mutant alleles, which were used for further functional studies.

### The subcellular localization of Ktub in *Drosophila* eye is light-dependent

**Figure 2:**
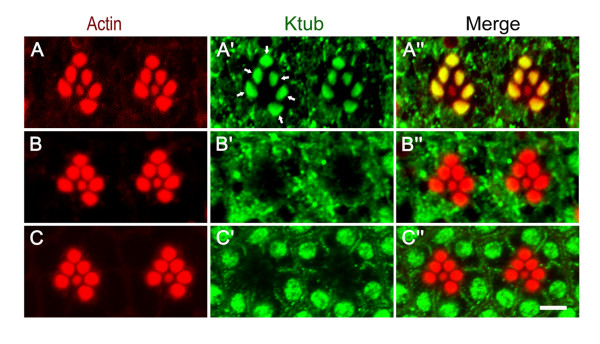
**Expression of Ktub in *Drosophila *photoreceptor cells. ***Drosophila* photoreceptor cells were stained with rhodamine-phalloidin and anti-Ktub antibody. When flies were reared under dark conditions, the majority of Ktub appeared in the rhabdomere domain (**A**’, arrows). The subcellular localization of Ktub is light sensitive. When flies were removed from the dark and immediately dissected in the light, the Ktub became to localize in the cytoplasm of photoreceptor cells (**B**’). When flies were exposed to light (500 lux) for 12 hours, the majority of Ktub appeared in the nucleus with some in the cytoplasm (**C**’). **A**, **B** and **C** were stained with rhodamine-phalloidin (red) A’, B’ and C’ were stained with anti-Ktub antibody (green). **A**”, **B**” and **C**” are merged images. The scale bar is 10 μm.

To study the function of Ktub in phototransduction and retinal degeneration, we generated antibodies against Ktub protein. The full length of Ktub recombinant protein was expressed using bacteria, and purified using SDS-PAGE. The purified protein was injected into rabbits to generate anti-Ktub antibody. Western blot analysis revealed that anti-Ktub antibody recognized a band at 50 kDa (Additional file 2: Figure S2A). The 50 kDa band disappeared when anti-Ktub antibody was pre-incubated with Ktub recombinant protein. These results indicate that the anti-Ktub antibody produced in this experiment is specific to Ktub protein (Additional file 2: Figure S2A). We also used this antibody to probe *Drosophila* S2 cells. When S2 cells incubated with anti-Ktub antibody, the Ktub protein appeared primarily in the nucleus (Addtional file 2: Figure S2B). When S2 cells were probed with anti-Ktub antibody, which has been pre-incubated with Ktub recombinant protein, no nuclear signals were detected. This result further indicates the specificity of anti-Ktub antibody generated in this study. To further study the function of Ktub in *Drosophila* photoreceptor cells, the adult wild type eye was stained with anti-Ktub antibody. When wild type flies were reared in the dark, the Ktub primarily appeared in the rhabdomere domain of photoreceptors R1 to R6 (Figure 2A’). No obvious Ktub signals were detected in the R7 rhabdomere, suggesting its specific function in the photoreceptors R1 to R6. When the dark-reared wild type flies were exposed to light for a few minutes, the rhabdomere localization disappeared, and the Ktub became localized in the cytoplasm instead (Figure 2B’). Under normal 12 hours light/12 hours dark conditions, the Ktub was mainly localized at the nucleus, with some in the cytoplasm of the photoreceptor cells (Figure 2C’). The specific cellular localization of Ktub in the photoreceptor cells between light and dark conditions suggesting its important function in phototransduction.

### Knockdown *ktub* expression resulted in light-dependent retinal degeneration

**Figure 3:**
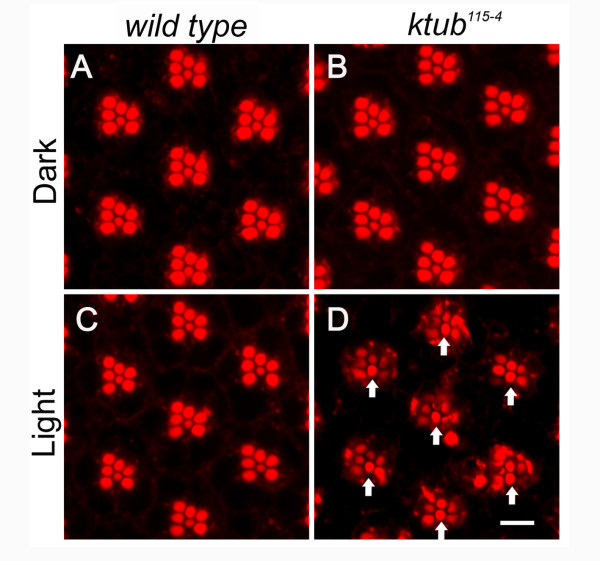
**Light-induced retinal degeneration in *ktub *mutant.** Young adults flies (<1 day) were reared in the dark (**A**, **B**), or under constant light (500 lux) (**C**, **D**) for 6 days. Confocal images indicate that photoreceptor cells arrayed as a typical trapezoid, and the rhabdomere appeared as an oval shape in the wild type and *ktub* mutant in dark condition (**A**, **C**). When flies were reared under constant light for 6 days, wild type photoreceptor cells remained normal (**B**), but the *ktub* mutant photoreceptor cells showed significant degeneration (**D**). The photoreceptor R7 was not affected, and remained as in the wild type (**D**, arrows). The scale bar is 5 μm.

**Figure 4:**
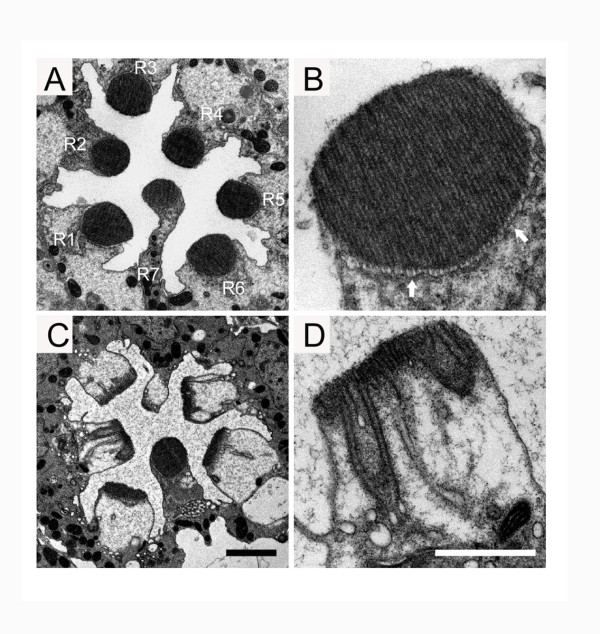
**TEM revealed retinal degeneration in *ktub *mutant.** Transmission electron micrographs indicated the photoreceptor cells of wild type and *ktub* flies raised under constant light for 6 days. Figures show one ommatidium of the wild type (**A**) and the *ktub* mutant retina (**C**). Higher magnification shows single rhabdomere in the wild type (**B**) and the *ktub* mutant (**D**). In the wild type, the rhabdomeres were organized as a regular array of microvilli. The plasma membrane at the base of the rhabdomere appeared as a catacomb-like extracellular space (**B**, arrows). In the *ktub* mutant, the rhabdomeres displayed short microvilli, distorted catacomb-like structures at the base, and curtains of microvillar membranes involuting into the photoreceptor cell (**C**, **D**). The photoreceptor R7 as not affected, and remained as in the wild type. The scale bar is 2 μm (**A**, **C**) and 1 μm (**B**, **D**).

Previous studies have found that mutation of mouse *tubby* caused late-onset and light-dependent retinal degeneration [47]. In this study, we examined whether mutation of *Drosophila ktub* causes retinal degeneration. To test this hypothesis, we reared wild type and *ktub* mutant flies under constant light (500 lux) or dark for 6 days. We dissected and stained the eyes with rhodamine–phalloidin to observe whether loss of *ktub* leads to retinal degeneration. Confocal images revealed that photoreceptor cells in wild type fly were organized in a typical trapezoid structure for both dark and light conditions (Figure 3A, C). In *ktub* mutant flies, the rhabdomeres remained intact in flies reared in the dark conditions (Figure 3B). However, the retinal degeneration become evident in photoreceptors R1 to R6 in *ktub* mutant flies which were reared in light conditions for 6 days (Figure 3D). Generally, the structure of the rhabdomeres appeared loose and lost their integrity when compared to the wild type (Figure 3C, D). The photoreceptor R7 was normal in *ktub* mutants. This observation is consistent with the expression of Ktub only found in photoreceptors R1 to R6 (Figure 2A’). To further investigate the function of Ktub in retinal degeneration, a transmission electron microscope was used to examine the ultrastructure of the photoreceptor cells in both wild type and *ktub* mutant flies. In light-reared conditions, the wild type photoreceptor cells consisted of regular ommatidia arrays. In a tangential section, seven photoreceptor cells were found in an ommatidium. Each photoreceptor cell had a distinguished photosensitive structure, the rhabdomere, which is organized by microvillar structures (Figure 4A, B). When *ktub* mutant was reared in light conditions for 6 days, the rhabdomere in photoreceptor R1 to R6 displayed short microvilli and distorted catacomb-like structures at the microvillar base. In addition, we often found that involution of the microvillar membrane into the photoreceptor cell in these mutant cells (Figure 4C, D). These observations suggest that *ktub* plays a critical role in maintaining rhabdomere integrity during phototransduction.

### *Drosophila ktub* participates in rhodopsin endocytosis

**Figure 5:**
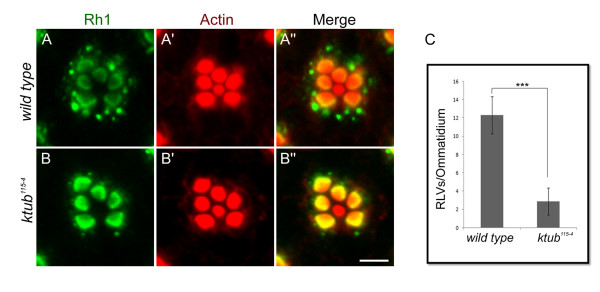
***ktub *participates in light-induced rhodopsin endocytosis.** Confocal images reveal rhodopsin localization in the photoreceptor cells of the wild type and *ktub* mutants. The flies received three hours of light stimulation and then investigated the endocytosis of rhodopsin in the photoreceptor cells. In the wild type (**A**), a significant amount of RLVs appeared in the cytoplasm of photoreceptor cells. In the *ktub* mutants (**B**), the majority of rhodopsin remained in the rhabdomere, with only few RLVs appearing in the cytoplasm of photoreceptor cells. Statistical analysis indicates the number of rhodopsin vesicles in the cytoplasm was significantly different in wild type and *ktub* mutants (three experiments, *Student’s t-test* *** indicating p < 0.001). The scale bar is 10 μm.

Previous research has shown that blocking endocytosis causes intensive retinal degeneration [48]. To determine whether the retinal degeneration in *ktub* mutant is caused by the blocking of endocytosis, this study analyzes the Rh1 endocytosis in flies containing a mutation in the *ktub* gene. When dark-reared wild type received three hours of light stimulation, a significant amount of the Rh1-immunopositive large vesicles (RLVs) were found in the cytoplasm (Figure 5A). In *ktub* mutants, however, the Rh1 was mainly localized in the rhabdomeric domain, and only a few RLVs were found in the cytoplasm (Figure 5B). The failure to completely block the Rh1 endocytosis in *ktub* mutant may be due to the present of short form of Ktub. Quantitative analysis showed the number of RLVs in the wild type and *ktub* mutant were significantly different suggesting an important role of *ktub* gene in mediating rhodopsin endocytosis (Figure 5C).

### *ktub* rescues *norpA* mediated endocytosis and retinal degeneration

**Figure 6:**
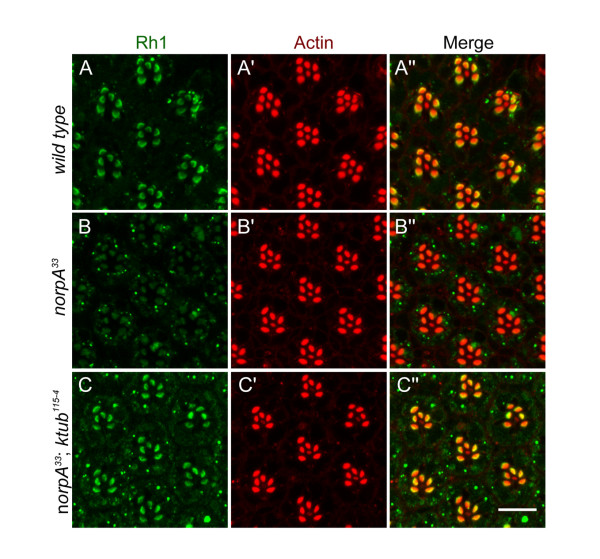
**Loss of *ktub *blocked *norpA*-mediated rhodopsin endocytosis.** Confocal images show the ability of rhodopsin endocytosis in wild type (**A**), *norpA*^*33*^ (**B**) and *norpA*^*33*^*/ktub*^*115-4*^double mutants (**C**). Flies were reared in the dark and then followed by light exposure (500 lux) for 24 hours. After light exposure, some RLVs were detected in the cytoplasm of the wild type photoreceptor cells (**A**). The *norpA*^*33*^ mutants exhibited massive RLVs in the cytoplasm of photoreceptor cells (**B**). However, the massive RLVs were rescued by loss of *ktub* in *norpA*^*33*^ mutants (**C**). **A**, **B** and **C** were stained with Anti-Rh1antibody (green), and **A**’, **B**’ and **C**’ were stained with rhodamine-phalloidin (red).

**Figure 7:**
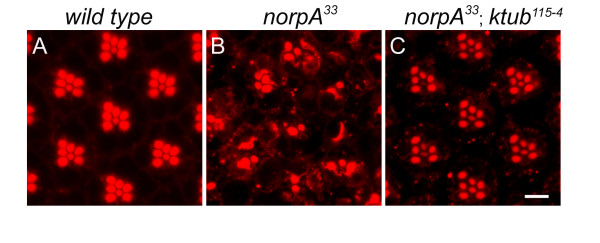
**Loss of *ktub *blocked *norpA*-mediated retinal degeneration.** Confocal images show photoreceptor cells stained with rhodamine-phalloidin in wild type (**A**), *norpA*^*33*^ (**B**) and *norpA*^*33*^*/ ktub*^*115-4*^ double mutants (**C**). Wild type photoreceptor cells were arrayed as a typical trapezoid in an ommatidum (**A**). The majority of photoreceptor cells were degenerated in *norpA*^*33*^mutant (**B**). In *norpA*^*33*^*/ktub*^*115-4*^double mutants, the retinal degeneration in *norpA*^*33*^mutant was rescued by loss of *ktub* gene (**C**). The scale bar is 10 μm.

*Drosophila norpA* is the eye-specific phospholipase C gene. Mutation of *norpA* causes massive internalization of rhodopsin from rhabdomere to the cell body [49]. To further investigate the role of *ktub* in endocytosis, this study examines whether *ktub* blocks *norpA*-mediated endocytosis. In wild type flies, the localization of Rh1 occurred mainly in the rhabdomere after 24 hours of light treatment (Figure 6A). In *norpA* mutants, the majority of rhodopsin disappeared in the rhabdomere, but formed RLVs in the cytoplasm after light stimulation (Figure 6B). In contrast, the majority of Rh1 in the *ktub* and *norpA* double mutants remained in the rhabdomere which was same as found in the wild type (Figure 6C). To further investigate the role of the *ktub* on *norpA* mediated retinal degeneration, wild type and mutant flies were treated with constant-light for 6 days to determine their retinal morphology. The wild type retina contains highly organized ommatidia; each ommatidium contains seven photoreceptor cells in a tangential section. The photosensitive structures, the rhabdomeres faced each other and arranged as a typical trapezoid (Figure 7A). The organization of photoreceptor cells in *norpA* mutants was severely distorted. Most of the rhabdomeres disappeared after 6 days of light stimulation (Figure 7B). In *ktub* and *norpA* double mutants, the organization of photoreceptor cells was almost same as in the wild type. The rhabdomeres appeared distinct and were arrayed as a typical trapezoid (Figure 7C). These observations demonstrate that the *norpA* mutant phenotype can be rescued by loss-of-function *ktub* allele suggesting that an important function of *ktub* in mediating rhodopsin endocytosis.

### Tubby domain is critical for mediating rhodopsin endocytosis

**Figure 8:**
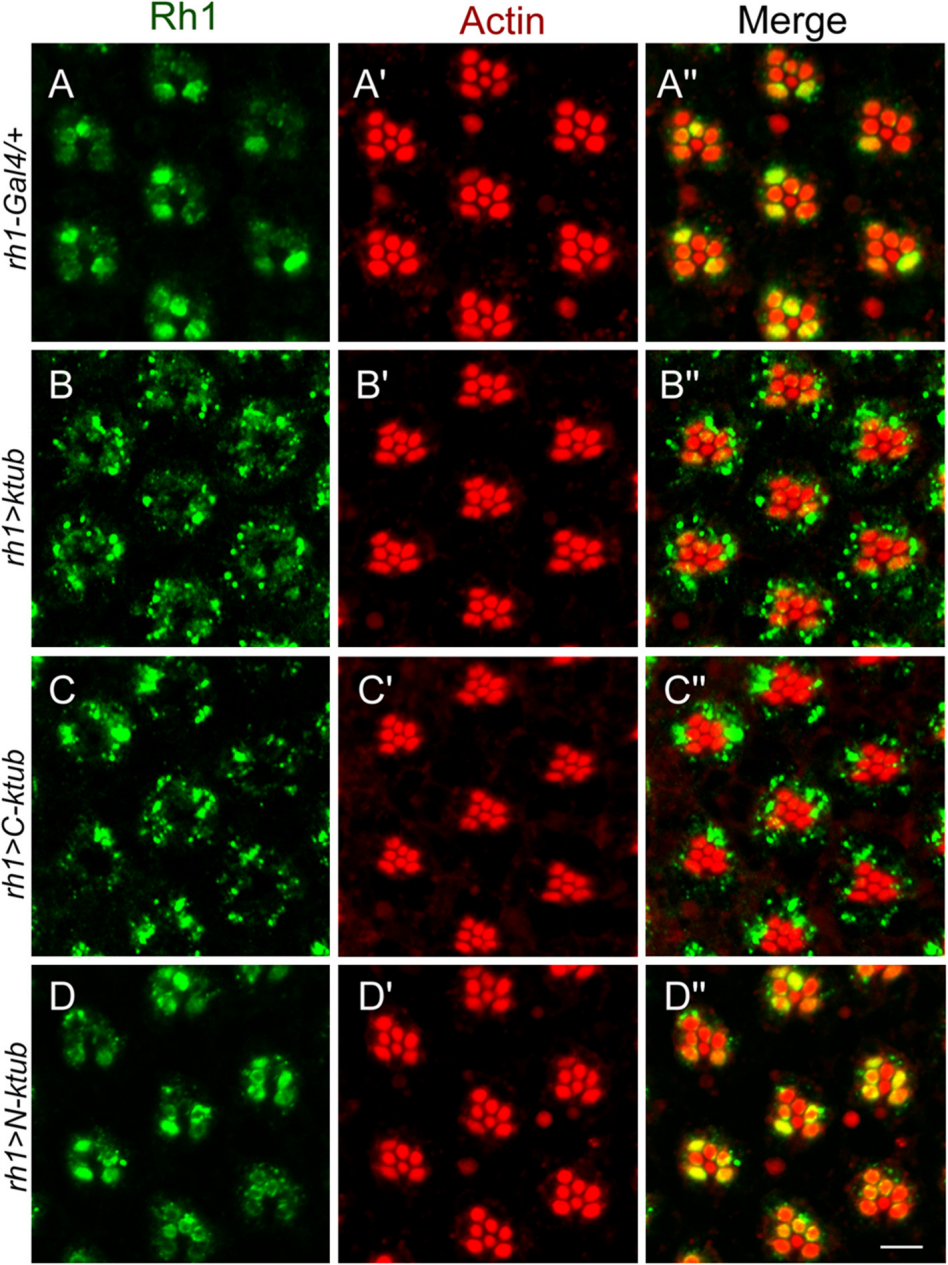
**Tubby domain of Ktub is critical for mediating rhodopsin endocytosis.** Confocal images show Rh1 endocytosis in photoreceptor cells. Flies received light stimulation for three hours and then processed for immunostaining with anti-Rh1 antibody and rhodamine-phalloidin. In wild type (**A**), some RLVs appeared in the cytoplasm of photoreceptor cells. In *rh1 > ktub* flies, massive RLVs appeared in the cytoplasm of photoreceptor cells (**B**). *rh1 > C-ktub* flies, many RLVs appeared in the cytoplasm of photoreceptor cells (**C**). In *rh1 > N-ktub* flies, only a few RLVs appeared in the cytoplasm of photoreceptor cells (**D**). The scale bar is 10 μm.

To further investigate the molecular mechanism of Ktub protein in endocytotic pathway, transgenic flies expressing different *ktub* deletion constructs were prepared to identify the specific protein domain involved in rhodopsin endocytosis. To achieve this goal, we generated three transgenic flies which express the full-length *(UAS-Flag-ktub*), the N-terminus (*UAS- Flag-N-ktub*) and the C-terminus (*UAS- Flag-C-ktub*) of the Ktub protein. Their ability to mediate rhodopsin endocytosis was tested by activating these transgenic flies with the photoreceptor specific driver, the *rh1-Gal4*. In control experiment, we crossed *rh1-Gal4* flies into wild type and found the rhodopsin was mainly localized in the rhabdomere after 3 hours of light activation (Figure 8A). Under the same conditions, the rhodopsin has formed the RLVs and been moved into the cytoplasm of the photoreceptor cells in *rh1-Gal4/UAS-Flag-ktub* (*rh1 > ktub*) flies (Figure 8B). When the N-terminus of Ktub protein was deleted in *rh1-Gal4/UAS-Flag-C-ktub* (*rh1 > C-ktub*) flies, the RLVs were also found in the cytoplasm, suggesting the endocytosis of rhodopsin into the cytoplasm of the photoreceptor cells (Figure 8C). When the C-terminus of Ktub was deleted in *rh1-Gal4/UAS-Flag-N-ktub* (*rh1 > N-ktub*) flies, few RLVs were found in the photoreceptor cells, suggesting the suppression of endocytotic process (Figure 8D). These results suggest that the C-terminus Tubby domain is required for mediating rhodopsin endocytosis.

## Discussion

The results of this study demonstrate that *Drosophila ktub* plays an important role in mediating Rh1 endocytosis. Immunocytochemical analysis reveals that Ktub is primarily located in the rhabdomeric domain in the dark. When the eye was exposed to light, the Ktub protein was immediately translocalized from the rhabdomere to the cytoplasm of the photoreceptor cells. This study further demonstrated that the subcellular expression pattern of the Ktub is correlated with Rh1 expression, which is also internalized upon light stimulation. The correlation of Ktub and Rh1 expression in photoreceptor cells suggests that these two molecules play similar roles during phototransduction. To determine whether Ktub regulates Rh1 internalization, we generated *ktub* mutants and assayed the role of Ktub in Rh1 endocytosis. Light-reared *ktub* mutant flies had significantly fewer RLVs in the cytoplasm of the photoreceptor than the wild type. In addition, light-stimulated *ktub* mutant flies also exhibited a severe retinal degeneration phenotype. To further demonstrate the role of *ktub* in endocytosis, we examined its ability to suppress *norpA*-mediated endocytosis. Results clearly demonstrate that *ktub* mutant blocked *norpA*-mediated endocytosis and retinal degeneration. Previous research has intensively studied the functions of Tub proteins in vertebrate systems. Several studies have shown that Tub and Tulp proteins are involved in retinal degeneration, obesity, and protein trafficking [19]. Studies also show that retinal degeneration in tubby mice is regulated by caspase-3 mediated pathway [47]. Currently, we do not know whether the retinal degeneration in *ktub* mutant is mediated by caspase-3 mediated pathway. However, previous studies show that the retinal degeneration in *norpA* mutant is not regulated by caspase mediated pathway [50]. The results in this study find that *ktub* mutant could rescue *norpA* mutant phenotype suggesting that the retinal degeneration in *ktub* mutant is not through caspase mediated pathway. Thus, it will be interested to study the molecular mechanism of Ktub in mediated retinal degeneration. This study shows that Ktub protein was mainly localized in the rhabdomeric microvilli of dark-reared flies (Figure 2). When flies were exposed to the light, the Ktub proteins translocated from the rhabdomere to the cytoplasm of the photoreceptor cells, suggesting that the function of Ktub is light-dependent (Figure 2). In *Drosophila* eyes, the photosensitive protein Rh1 is localized in the rhabdomeric microvilli in dark-reared flies. When flies receive light stimulation, rhodopsin kinase phosphorylates the Rh1 at its C-terminus [51,52] and then internalizes into the cytoplasm of the photoreceptor cells (Figure 5 and [38] ). Arrestin 1 and arrestin 2 are required for Rh1 endocytosis upon light stimulation [37,53]. In the *arrestins* mutant, Rh1 remains in the active stage in the phototransduction cascade. The consequence of active Rh1 accumulation causes photoreceptor cells to undergo light-dependent retinal degeneration [54]. Previous studies have revealed massive Rh1 endocytosis in *norpA* mutants, and that the Rh1 endocytosis in *norpA* mutant is blocked by the *shi*^*ts*^ mutant allele [38,55]. The current study reveals that the formation of RLVs in *ktub* mutant is less than that in wild type upon light stimulation (Figure 5). The *ktub/norpA* double mutant also suppressed Rh1 endocytosis and retinal degeneration, further supporting that Ktub regulates Rh1 endocytosis (Figure 6). In mice, Dynamin-1 is colocalized with TULP1 in the outer plexiform layer and the inner segments of retina [21]. Biochemical analysis demonstrates that Dynamin-1 binds directly to TULP1[21]. Dynamin-1 is a major component of vesicle formation in receptor mediated endocytosis, synaptic vesicle recycling, and vesicle trafficking in and out of the trans-Golgi network [56-58]. This study shows that Ktub has a similar function to Dynamin-1 in blocking *norpA*-mediated massive Rh1 endocytosis. Although this study does not provide evidence showing a direct interaction between Ktub and *Drosophila* Dynamin, the idea that Ktub may bind to *Drosophila* Dynamin is consistent with the observation that Tub protein binds to Dynamin and regulates vesicle transport in mice [21]. In addition, the TUB-1 protein in *C. elegans* controls fat storage through the RAB-7 mediated endocytosis pathway [3]. Previous studies support the function of Ktub in mediating the endocytotic pathway. Further research should test whether Ktub also binds to Dynamin or whether it mediates the endocytotic pathway through Rab-7.

To determine which domain of Ktub protein is involved in endocytosis, this study uses Ktub deletion constructions in which either the N-terminus or C-terminus of Ktub protein was deleted. This study also tests which constructs failed to mediate Rh1 endocytosis in photoreceptor cells. Results show that the deletion of the C-terminus in the Ktub protein blocked light-induced Rh1 endocytosis (Figure 8). Structural analysis indicates that the Tubby C-terminal domain binds to double strand DNA and its N-terminal domain activates transcription [18]. Previous studies have also shown an unconventional secretion of Tubby and Tulp1, indicating that they function as phagocytosis ligands for retinal pigment epithelium and macrophage phagocytosis [24,25]. Further investigation reveals that Tubby and Tulp1 act as bridging molecules, as their N-terminal region functions as an MreTK- binding domain and the C-terminal region functions as a phagocytosis prey-binding domain [26]. This study shows that the deletion of the Ktub C-terminus blocked Rh1 endocytosis, suggesting that the C-terminus may contain an important domain for mediating endocytosis. It is worth further investigation which specific peptide sequence is involved in mediating endocytosis. Because of the large fragment of deletion, it cannot be ruled out the possibility that the results from the deletion constructs are not physiological. Although the role of the C-terminus of Ktub protein in endocytosis is not completely clear, this study provides valuable information to further investigate the function of Ktub in endocytosis. Taken together, this study provides substantial support for the function of *Drosophila ktub* in mediating Rh1 endocytosis and retinal degeneration under light-dependent conditions.

## Conclusions

This study examined the function of *Drosophila ktub* gene in phototransduction. Immunocytochemical studies showed the subcellular localization of Ktub in photoreceptor cells is light-dependent. In *ktub* mutants, the rhodopsin endocytosis is blocked under light stimulation. We also observed a retinal degeneration phenotype in *ktub* mutants. Using deletion constructs, we found the C-terminus of Ktub is required for Rh1 endocytosis. Taken together, these results delimit the novel function of Ktub in Rh1 endoytosis and retinal degeneration.

## Competing interests

The authors declare that they have no competing interests.

## Authors’ contributions

SFC, YCT and SSF designed the experiments. SFC performed the genetic screening and all the experiments. YCT helped the genetic experiment. SSF wrote the manuscript. All authors read and approved the final manuscript.

## Supplementary Material

Additional file 1 **Figure S1.** Sequence alignments of*Drosophila* Ktub-long (Ktub-L) form and short (Ktub-S) form. Two peptide sequences were aligned using Expasy. The peptide sequence between long form and short form match perfectly with the exception of an additional 17 amino acids in the N-terminus of the long form protein. The blue line indicates the Tubby domain. Click here for file

Additional file 2 **Figure S2.** Determining the specificity of anti-Ktub antibody. Western blot analysis reveals that anti-Ktub antibody recognizes a 50 kDa protein (A, arrow). When antibody was preincubated with Ktub recombinant protein, the 50 kDa band disappeared (A). Confocal images show Ktub expression in the *Drosophila* S2 cells (B). When *Drosophila* S2 cells were probed with anti-Ktub antibody, the antibody detected a nuclear signal in the S2 cells. The nuclear signal disappeared when antibody was preincubated with Ktub recombinant protein. Propidium iodide (PI) stains for nucleus (red). The scale bar is 10 μm. Click here for file
