# Supplementary figures and images for: Drosophila king tubby (ktub) mediates light-induced rhodopsin endocytosis and retinal degeneration
Source: J Biomed Sci. 2012 Dec 10;19(1):101. doi: 10.1186/1423-0127-19-101 (PMC3541268; doi:10.1186/1423-0127-19-101)

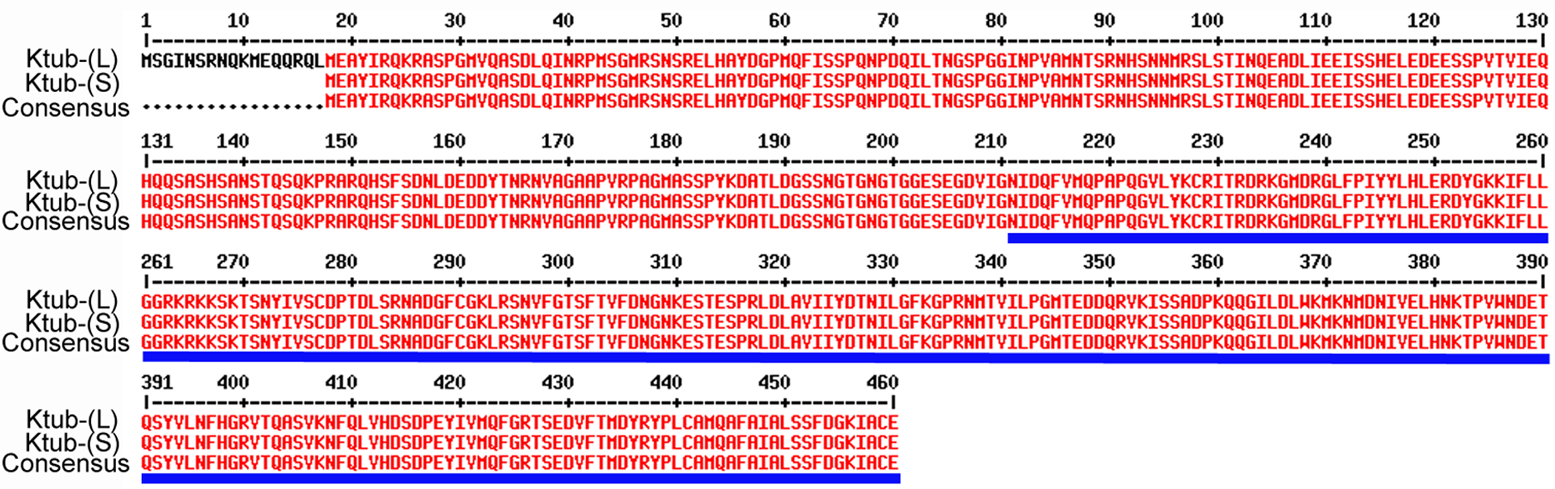

Supplement: Additional file 1 — Figure S1. Sequence alignments ofDrosophila Ktub-long (Ktub-L) form and short (Ktub-S) form. Two peptide sequences were aligned using Expasy. The peptide sequence between long form and short form match perfectly with the exception of an additional 17 amino acids in the N-terminus of the long form protein. The blue line indicates the Tubby domain. [file 1423-0127-19-101-S1.tiff]

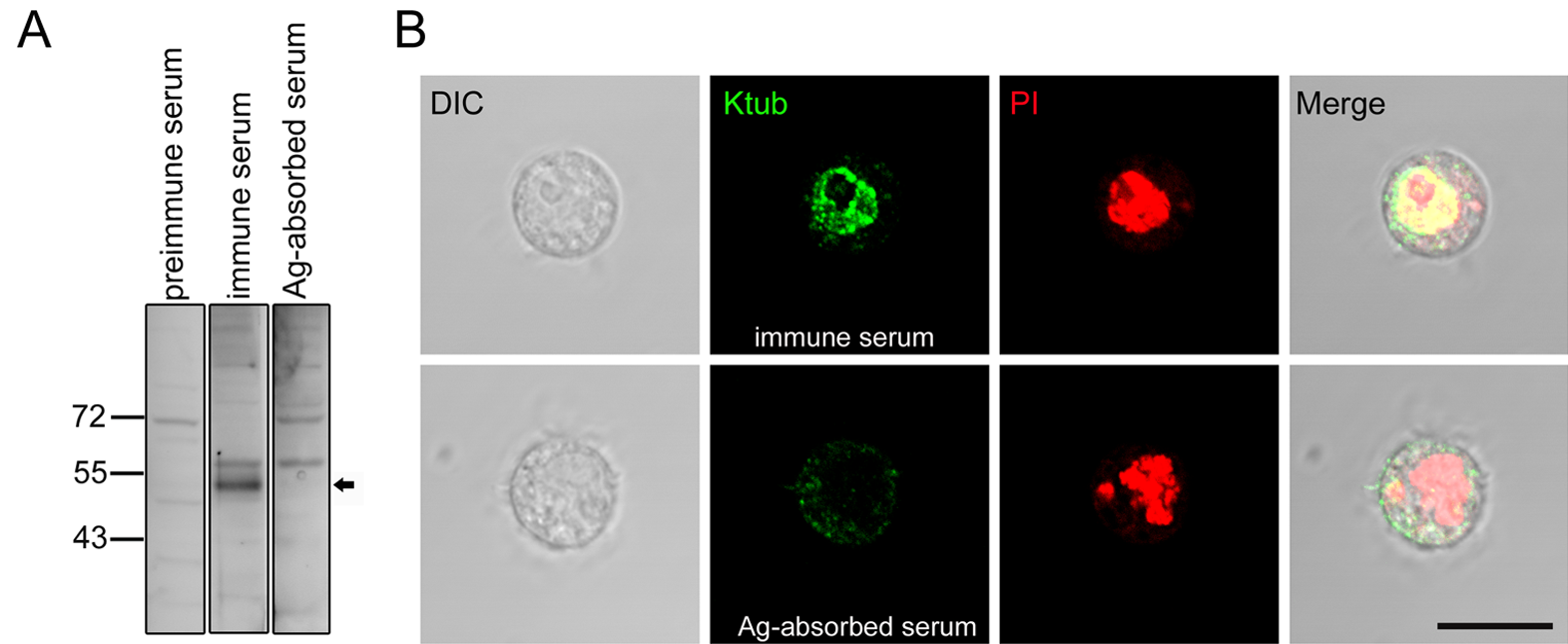

Supplement: Additional file 2 — Figure S2. Determining the specificity of anti-Ktub antibody. Western blot analysis reveals that anti-Ktub antibody recognizes a 50 kDa protein (A, arrow). When antibody was preincubated with Ktub recombinant protein, the 50 kDa band disappeared (A). Confocal images show Ktub expression in the Drosophila S2 cells (B). When Drosophila S2 cells were probed with anti-Ktub antibody, the antibody detected a nuclear signal in the S2 cells. The nuclear signal disappeared when antibody was preincubated with Ktub recombinant protein. Propidium iodide (PI) stains for nucleus (red). The scale bar is 10 μm. [file 1423-0127-19-101-S2.tiff]
